# Disparities in breast screening, stage at diagnosis, cancer treatment and the subsequent risk of cancer death: a retrospective, matched cohort of aboriginal and non-aboriginal women with breast cancer

**DOI:** 10.1186/s12913-019-4147-5

**Published:** 2019-06-14

**Authors:** David Banham, David Roder, Dorothy Keefe, Gelareh Farshid, Marion Eckert, Natasha Howard, Karla Canuto, Alex Brown, for the CanDAD Aboriginal Community Reference Group and other CanDAD investigators

**Affiliations:** 1 Wardliparingga Aboriginal Research Unit, South Australian Health and Medical Research Institute, North Terrace, Adelaide, SA 5000 Australia; 2 School of Health Sciences, Cancer Research Institute, University of South Australia, North Terrace, Adelaide, SA 5000 Australia; 3 University of South Australia, North Terrace, Adelaide, SA 5000 Australia; 4 SA Cancer Services, SA Health, Hindmarsh Square, Adelaide, SA 5000 Australia; 5 Faculty of Health Sciences, University of Adelaide, North Terrace, Adelaide, SA 5000 Australia; 6 School of Nursing and Midwifery, University of South Australia, North Terrace, Adelaide, SA 5000 Australia; 7 School of Health Sciences, Aboriginal Health Research Group, University of South Australia, North Terrace, Adelaide, SA 5000 Australia

**Keywords:** Breast cancer, Cancer screening, Cancer treatment, Aboriginal, Indigenous, Survival

## Abstract

**Background:**

Australia’s Aboriginal and Torres Strait Islander women have poorer survival and twice the disease burden from breast cancer compared to other Australian women. These disparities are influenced, but not fully explained, by more diagnoses at later stages. Incorporating breast screening, hospital and out of hospital treatment and cancer registry records into a person-linked data system can improve our understanding of breast cancer outcomes. We focussed one such system on a population-based cohort of Aboriginal women in South Australia diagnosed with breast cancer and a matched cohort of non-Aboriginal women with breast cancer. We quantify Aboriginal and non-Aboriginal women’s contact with publicly funded screening mammograms; quantify exposure to a selection of cancer treatment modalities; then assess the relationship between screening, treatment and the subsequent risk of breast cancer death.

**Methods:**

Breast cancers registered among Aboriginal women in South Australia in 1990–2010 (*N* = 77) were matched with a random selection of non-Aboriginal women by birth and diagnostic year, then linked to screening records, and treatment 2 months before and 13 months after diagnosis. Competing risk regression summarised associations of Aboriginality, breast screening, cancer stage and treatment with risk of breast cancer death.

**Results:**

Aboriginal women were less likely to have breast screening (OR = 0.37, 95%CIs 0.19–0.73); systemic therapies (OR = 0.49, 95%CIs 0.24–0.97); and, surgical intervention (OR = 0.35, 95%CIs 0.15–0.83). Where surgery occurred, mastectomy was more common among Aboriginal women (OR = 2.58, 1.22–5.46). Each of these factors influenced the risk of cancer death, reported as sub-hazard ratios (SHR). Regional spread disease (SHR = 34.23 95%CIs 6.76–13.40) and distant spread (SHR = 49.67 95%CIs 6.79–363.51) carried more risk than localised disease (Reference SHR = 1). Breast screening reduced the risk (SHR = 0.07 95%CIs 0.01–0.83). So too did receipt of systemic therapy (SHR = 0.06 95%CIs 0.01–0.41) and surgical treatments (SHR = 0.17 95%CIs 0.04–0.74). In the presence of adjustment for these factors, Aboriginality did not further explain the risk of breast cancer death.

**Conclusion:**

Under-exposure to screening and treatment of Aboriginal women with breast cancers in South Australia contributed to excess cancer deaths. Improved access, utilisation and quality of effective treatments is needed to improve survival after breast cancer diagnosis.

## Background

Australia’s Aboriginal and Torres Strait Islander women have poorer breast cancer survival [1] and twice the disease burden from breast cancer compared to other Australian women [2]. These disparities in outcomes are influenced, but not fully explained [3], by relatively more diagnoses at later tumour stages [4].

Early detection of breast cancers using mammographic screening can improve survival outcomes [5–8] but disparities in participation are evidenced internationally on the basis of ethnicity [9] and Indigeneity, for example among Maori women in New Zealand [5, 10]. After breast cancer diagnosis, disparities in cancer treatment are also recorded in the US, particularly between African American and Hispanic women compared to white Americans [9]. In New Zealand too, chemo and radio therapies were less frequent among Maori than other women [11, 12] as was surgical treatment [13]. Further, when surgery occurred, Maori women were more likely to have a mastectomy rather than breast conserving surgery [14].

A review of Australian breast cancer screening and treatment patterns concluded there was consistent evidence of lower screening [15–17] and evidence of lower rates of hospital treatment for breast cancers among Aboriginal and Torres Strait Islander women [17]. Different rates of surgery have been reported by state and territory jurisdictions with Aboriginal and Torres Strait Islander women receiving comparable treatment to non-Indigenous women in Queensland [18] but significantly less surgical intervention in New South Wales (NSW) [19]. A national analysis of women participating in breast screening found, however, that Aboriginal and Torres Strait Islander women were more likely than other women to undergo mastectomy rather than breast conserving surgery [6, 16].

To improve understanding of Aboriginal South Australians’ cancer outcomes, an advanced cancer data system was developed incorporating person-linked registry, hospital, Pharmaceutical Benefits Scheme and Medicare Benefit Schedule records within a wider Cancer Data and Aboriginal Disparities (CanDAD) project [20]. CanDAD has previously described the role of Aboriginality, cancer stage at diagnosis and broad treatment modalities in cancer outcomes for all cancer types collectively [3, 21]. Female breast screening service records [6, 16] were also available and facilitated an additional step in quantifying the clinical pathway for the subgroup of Aboriginal women diagnosed with breast cancer [22]. This will assist with developing an improved national understanding of breast cancer outcomes. Within South Australia, it will help identify any unmet capacity to benefit from informed, tailored service responses.

In pursuing three goals our study focusses on a population-based cohort of Aboriginal women in South Australia diagnosed with breast cancer and a matched cohort of non-Aboriginal women. Firstly, we examine differences in aspects of Aboriginal and non-Aboriginal women’s contact with BreastScreen South Australia (BSSA), the government agency providing screening mammograms, by quantifying the exposure to screening through BSSA. We then quantify disparate exposure to a selection of cancer treatment modalities before assessing the relationship between Aboriginality and stage at diagnosis, screening, treatment and the subsequent risk of death from breast cancer.

## Methods

A retrospective cohort study involving Aboriginal women in South Australia diagnosed with breast cancer between 1990 and 2010 and a randomly selected cohort of non-Aboriginal women with breast cancer, matched by years of birth and diagnosis.

### Study governance

CanDAD’s Aboriginal Community Reference Group governance ensured alignment of the study protocol with the principles of the South Australian Aboriginal Health Research Accord [23].

### Study design and participants

A retrospective cohort of all breast cancer cases diagnosed among female, Aboriginal South Australians in the period 1990 to 2010 (*N* = 77) matched one to one with a random selection of breast cancer cases among non-Aboriginal females by birth year and diagnosis year.

### Data sources and measurements

De-identified breast cancer data were provided by the South Australian Cancer Registry (SACR), a population-based registry coding diagnoses according to the International Classification of Diseases for Oncology [24] and further linked to ICD-10 coded causes and dates of death. Cancer stage at diagnosis was described using Surveillance, Epidemiology, and End Results Program summary staging [25] as: *localised -* confined to tissue of origin; *regional -* invaded adjacent tissue or regional nodes; *distant -* spread to distant lymph nodes or other organs; and *unknown* stage where insufficient staging data were available. *Distant* and *unknown* stages were previously shown to have similar risk of cancer death in the cohorts [3] and were aggregated to ensure adequate cell sizes in our reporting.

SACR also records postal area at diagnosis, each of which is categorised by quintile of socio-economic disadvantage using the Australian Bureau of Statistics Census 2011 Index of Relative Socio-economic Advantage and Disadvantage (ISRAD) [26]. For this study, we further collapsed quintiles into two groups: Most disadvantaged - Quintile 1; and, All others - Quintiles 2 to 4.

Aboriginal status was established after cross-referencing SACR records with linked study datasets including SA-NT Datalink’s master linkage file, public hospital inpatient and clinical information systems data and death records [20]. The latter three collections each incorporate a person’s self-identification of Aboriginal status. We adopted that approach to avoid including false positives (classifying non-Aboriginal cases as Aboriginal) and biasing survival differences towards the null. While some false classification of Aboriginality may have persisted, we believe misclassification would have been rare and have little effect on comparisons by Aboriginal status.

South Australian women participating in the BSSA program from 1989 to 2010 were probabilistically linked with SACR breast cancer diagnoses [22]. Cohort cases were then classified as: not linked and therefore *not listed* on BSSA records; *listed* on BSSA records but with no screening history; and listed and *screened* by BSSA.

Three treatment modes were included: surgery, systemic therapies and radiotherapy. Surgery was classified as excision or destruction according to Australian standard classifications of procedure invasiveness [27] with two further subcategories of: partial mastectomy (procedures 3150000 and 3151500) and simple/subcutaneous mastectomy (31518xx, 31524xx). Person-linked hospitalisations across the period 1 July 1991 to 30 June 2013 and two months before and 13 months after the SACR recorded diagnosis month were sourced from the Integrated South Australian Activity Collection and Alice Springs Hospital in the Northern Territory [20, 21]. Systemic cancer therapies of antineoplastic and immune-modulating agents were sourced from the same hospital records and Pharmaceutical Benefits Scheme records dated from 1 July 2002. Radiotherapy notifications were also obtained from: the SACR; hospitalisations; and Medicare Benefit Schedule records dating from 1988 onwards.

### Outcomes

The primary outcome was survival time from cancer diagnosis to death from breast cancer or right censoring when the observation period ended on 31st December 2011, whichever occurred first.

### Statistical analysis

All analyses were performed within the Secure Unified Research Environment [28] using Stata 14 [29]. Bivariate associations between Aboriginality, socio-demographic (age and area level disadvantage) and cancer related variables were examined using conditional logistic regression and cross-tabulations. In assessing the adequacy of each stratum cell size we applied threshold rules [30] of five or more in sensitive areas focussed on medical issues and treatment and three or more on the less sensitive issue of being listed/not listed within administrative contacts [31]. Associations within each BSSA classification and treatment mode were cross-tabulated and Pearson’s Chi-Square Test used to highlight cells contributing to significant differences within strata (at *p* < 0.05). The adjusted odds of receiving treatment types were assessed concurrently by Aboriginal status and localised/non-localised stage at diagnosis using multilevel logistic regression analyses (Stata’s *melogit*) [32].

Multivariable analyses of the risk of breast cancer death are reported using sub-hazard ratio (SHR) estimates which accounted for competing risk from non-cancer mortality using Fine and Gray’s [33] method and Stata’s *stcrprep* with *stcox.* Baseline Model 1 included the main effects of Aboriginal status as exposure variable and stage at diagnosis (categorised as: localised disease; cancers with regional spread; and, those with advanced, distant spread or where spread was not determined) as moderator. Models 2 and 3 added BSSA listing and screening respectively. Model 4 included surgery, consisting of procedures for excision or destruction, and systemic therapy, involving antineoplastic or immune-modulating therapies, as treatment modes having significant influences on the risk of cancer death. Three surgical sub-categories of no, partial and simple/complete mastectomy were added in Model 5. Each model’s parsimony and fit to the cohort data were considered against Akaike Information Criterion (AIC) statistics [34]. Each Model’s adherence to the proportional hazards assumption was assessed by referring to Schoenfeld residuals [35].

## Results

The Aboriginal (*N* = 77) and non-Aboriginal breast cancer cohorts were equivalent on matching variables of year of birth and year of diagnosis. Table 1 shows Aboriginal women with breast cancer were more likely to be living in the most disadvantaged quintile (IRSAD Q1) with unadjusted OR = 8.15 (95%CI 3.84–17.31). They had a lower likelihood of formal contact with BSSA and were less likely again to have a breast screening history (OR = 0.52, 95%CIs 0.26–1.03 and OR = 0.37, 95%CIs 0.19–0.73 respectively). Aboriginal women were less likely to have records of hospitalisation for breast cancer and treatment by systemic therapies (OR = 0.49, 95%CIs 0.24–0.97). Surgical intervention was comparatively less frequent among Aboriginal women (OR = 0.35, 95%CIs 0.15–0.83) but more likely to involve simple, total mastectomy when it did occur (OR = 2.58, 95%CIs 1.22–5.46).

**Table 1:** Demographic distribution, tumour characteristics, comorbid conditions and cancer treatment types by Aboriginality

	Aboriginal cohort		Matched non-Aboriginal cohort			
	N	%	N	%	OR (unadjusted)	95% CIs
Total	77	100.0%	77	100.0%		
Age						
< 50 years	22	28.6%	20	26.0%		
50–69 years	41	53.2%	41	53.2%		
70+ years	14	18.2%	16	20.8%		
2011 IRSAD Disadvantage						
Most disadvantage Q1	48	62.3%	13	16.9%	8.15	3.84–17.31
Other Quintiles Q2 -Q4	29	37.7%	64	83.1%	1.00	Reference
Summary stage at diagnosis						
Localised	36	46.8%	47	61.0%	1.00	Reference
Regional	24	31.2%	24	31.2%	1.31	0.64–2.66
Distant/Unknown	17	22.1%	6	7.8%	2.87	0.92–9.01
BreastScreen SA (BSSA) contact						
Not listed with BSSA	31	40.3%	20	26.0%	1.00	Reference
Listed with BSSA, not screened	23	29.9%	16	20.8%	0.52	0.26–1.03
BSSA screened	23	29.9%	41	53.2%	0.37	0.19–0.73
Cancer treatment^a,b^						
Hospitalisation with cancer diagnosis	62	80.5%	71	92.2%	0.35	0.13–0.96
Systemic therapy	46	59.7%	58	75.3%	0.49	0.24–0.97
Surgery^*c*^	56	72.7%	68	88.3%	0.35	0.15–0.83
*Mastectomy*						
*Partial*	*20*	*26.0%*	*38*	*49.4%*	*1.00*	*Reference*
*Simple*	*34*	*44.2%*	*25*	*32.5%*	*2.58*	*1.22–5.46*
Radiotherapy	41	53.2%	51	66.2%	0.58	0.30–1.11

^a^Up to 2 months before and 13 months after month of diagnosis

^b^Categories are not mutually exclusive

^c^Surgery includes a small number of excisions not categorised as mastectomy

The cohort characteristics within selected BSSA classifications are summarised in Table 2. Aboriginal women aged less than 50 years of age represented about one quarter of cases (29%) but less than 10% of Aboriginal breast cancer cases listed with BSSA compared to 19% of non-Aboriginal cases. In particular, the age distribution of cohort members not listed by BSSA features included comparatively more and younger Aboriginal women than non-Aboriginal.

**Table 2:**
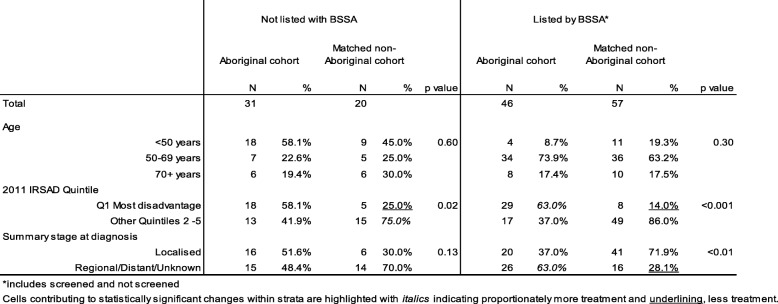
Characteristics of cohort cases listed and screened by BSSA

The characteristics of cohort cases receiving various treatments (Table 3) were consistently distributed across systemic therapies, surgery and radiotherapy with the exception of comparatively less frequent systemic therapy among localised breast cancers in Aboriginal women.

**Table 3:**
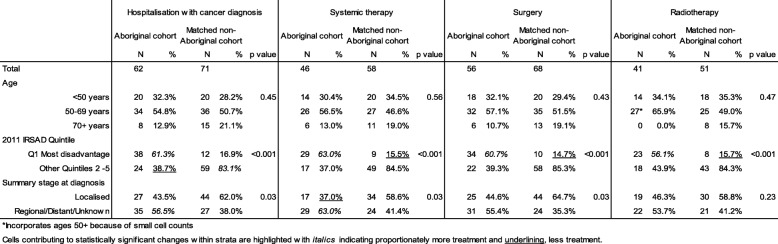
Characteristics of cohort cases receiving treatment

The simultaneous relationship between receipt of treatment, stage at diagnosis and Aboriginality is summarised in Table 4. Stage at diagnosis was not clearly predictive of treatment received. However, Aboriginality was associated with lower likelihood of hospitalisation with a principal diagnosis of cancer (OR = 0.25, 95%CIs 0.07–0.09), receiving systemic therapy (OR = 0.19, 95%CIs 0.05–0.75) and surgery (OR = 0.21, 95%CIs 0.06–0.75).

**Table 4:** Regression analysis relating treatment receipt, Aboriginality and stage at diagnosis

		OR (Adjusted)	95% CIs	z	p > |z|
Hospitalisation with cancer diagnosis					
	Aboriginal				
	No	1.00	Reference		
	Yes	0.25	0.07–0.90	−2.13	0.03
	Stage at diagnosis				
	Localised	1.00			
	Regional	1.07	0.08–13.57	0.05	0.96
	Distant/Unknown	1.30	0.14–12.47	0.23	0.82
Systemic therapy					
	Aboriginal				
	No	1.00	Reference		
	Yes	0.19	0.05–0.75	−2.37	0.02
	Stage at diagnosis				
	Localised	1.00	Reference		
	Regional	5.42	0.86–34.06	1.80	0.07
	Distant/Unknown	7.25	0.49–107.89	1.44	0.15
Surgery					
	Aboriginal				
	No	1.00	Reference		
	Yes	0.21	0.06–0.75	−2.42	0.02
	Stage at diagnosis				
	Localised	1.00			
	Regional	0.81	0.09–7.52	−0.18	0.85
	Distant/Unknown	0.53	0.06–5.03	−0.55	0.58
Radiotherapy					
	Aboriginal				
	No	1.00	Reference		
	Yes	0.56	0.27–1.17	−1.54	0.12
	Stage at diagnosis				
	Localised	1.00	Reference		
	Regional	2.34	0.75–7.28	1.47	0.14
	Distant/Unknown	0.99	0.24–4.01	−0.01	0.99

Tables 5 and 6 expresse the risk of breast cancer death using sub-hazard ratios (SHR). Baseline Model 1 indicates that Aboriginal women with breast cancer experienced a comparatively greater risk of cancer death than non-Aboriginal cases (SHR = 5.13, 95%CIs 1.22–21.66) with regional and distant/unknown staged disease at diagnosis also being associated with markedly higher risk of cancer death than localised disease (SHR = 14.21, 95%CIs 0.76–264.00 and SHR = 13.74, 95%CIs 1.77–106.94 respectively). Model 2 did not find BSSA listing to be a statistically significant protective factor against risk of cancer death. In examining the influence of completed BSSA screening however, Model 3 found a screening history with BSSA was associated with a reduced risk of cancer death, SHR = 0.13 (95%CIs 0.03–0.66). Further, the effect of stage at diagnosis remained but Aboriginality no longer significantly affected the risk of cancer death and was removed. On adding treatment modalities to Model 4, the effects of stage at diagnosis and breast screening on risk of cancer death continued while both exposure to systemic therapies (SHR = 0.06, 95%CIs 0.01–0.41) and surgical treatment (SHR = 0.17, 95%CIs 0.04–0.74) also showed independent associations with reduced risk of cancer death. On further disaggregating surgery into partial and simple mastectomy in Model 5, both showed an association with reduced risk of cancer death. Models 4 and 5 were considered to best fit the cohort data on the basis of their having the lowest AIC values observed. The assumption of proportional hazards was upheld in each of the models reported.

**Table 5:** Competing risk regression analysis for cancer survival among Aboriginal and matched non-Aboriginal cohorts, South Australia 1990–2010

	Model 1 - baseline				Model 2 - BSSA listed				Model 3 - BSSA screened			
	Subhazard Risk Ratio	95% CIs	z	p > |z|	Subhazard Risk Ratio	95% CIs	z	p > |z|	Subhazard Risk Ratio	95% CIs	z	p > |z|
Aboriginal												
No	1.00	Reference			1.00	Reference						
Yes	5.13	1.22–21.66	2.23	0.026	4.81	1.12–20.66	2.11	0.035				
Stage at diagnosis												
Local	1.00	Reference			1.00	Reference			1.00	Reference		
Regional	14.21	0.76–264.00	1.78	0.075	14.07	0.80–246.18	1.81	0.070	7.88	1.90–32.64	2.85	0.004
Distant or Unknown	13.74	1.77–106.94	2.50	0.012	13.79	1.67–113.85	2.44	0.015	17.68	3.93–79.61	3.74	0.000
Listed with BSSA												
No					1.00	Reference						
Yes					0.71	0.16–3.10	−0.46	0.647				
Screened by BSSA												
No									1.00	Reference		
Yes									0.13	0.03–0.66	−2.46	0.014
Systemic therapy												
No												
Yes												
Surgical treatment												
No												
Yes												
Mastectomy												
No mastectomy												
Partial												
Simple/ subcutaneous												
AIC	30.1				32.0				32.0

**Table 6:** Competing risk regression analysis for cancer survival among Aboriginal and matched non-Aboriginal cohorts, South Australia 1990–2010

	Model 4 - treated				Model 5 - treated (with mastectomy)			
	Subhazard Risk Ratio	95% CIs	z	p > |z|	Subhazard Risk Ratio	95% CIs	z	p > |z|
Aboriginal								
No								
Yes								
Stage at diagnosis								
Local	1.00	Reference			1.00	Reference		
Regional	34.23	6.76–173.40	4.27	0.000	48.81	3.63–655.42	2.93	0.003
Distant or Unknown	49.67	6.79–363.51	3.85	0.000	69.21	4.21–1138.25	2.97	0.003
Listed with BSSA								
No								
Yes								
Screened by BSSA								
No	1.00	Reference			1.00	Reference		
Yes	0.07	0.01–0.83	−2.11	0.035	0.05	0.01–0.40	−2.78	0.005
Systemic therapy								
No	1.00	Reference			1.00	Reference		
Yes	0.06	0.01–0.41	−2.88	0.004	0.05	0.01–0.21	−4.11	0.000
Surgical treatment								
No	1.00	Reference						
Yes	0.17	0.04–0.74	−2.36	0.018				
Mastectomy								
No mastectomy					1.00	Reference		
Partial					0.10	0.01–0.88	−2.08	0.038
Simple/ subcutaneous					0.23	0.06–0.86	−2.19	0.029
AIC	28.9				28.9			

## Discussion

This population-based study of Aboriginal and non-Aboriginal women with breast cancer, matched by years of birth and diagnosis, found that localised cancers were consistently associated with lower risk of death from breast cancer compared to those with more advanced spread at diagnosis. Our initial examination found this was accompanied by a much greater risk of cancer death among Aboriginal women compared to non-Aboriginal women of equivalent age and year of diagnosis. However, the level of risk associated with Aboriginality was mitigated by a history of breast screening through BSSA, a marker of reduced risk of cancer death. This continued to be the case in the presence of treatment with systemic therapies and surgical intervention, each of which significantly and independently reduced the risk of cancer death further. While finding no evidence of differential treatment effects by these factors for Aboriginal and non-Aboriginal cases, we observed clear differences in exposure to risk reducing factors. Specifically, there was a lack of contact with, and screening by, BSSA among Aboriginal cases relative to their matched, non-Aboriginal contemporaries. Moreover, there was less exposure to cancer treatment. Where surgery did take place, mastectomy rather than breast conserving surgery was more than twice as likely among Aboriginal women. In short, our results indicate that higher exposure to more advanced disease at diagnosis and lower exposure to breast screening and systemic/surgical treatments explained the higher risk of cancer death among similarly aged women with breast cancer, not Aboriginality as such.

Our findings are consistent with the wider breast screening literature that clearly indicates underutilisation of breast screening among ethnic minorities and indigenous populations [5, 9, 10]. Most specifically within the Australian context, the Australian Institute of Health and Welfare [15] report around 33% of Aboriginal and Torres Strait Islander and 53% of non-Indigenous women in the relevant age ranges participate in breast screening. Our results are closely aligned to this with BSSA screening histories noted among 30 and 53% of Aboriginal and non-Aboriginal cases respectively.

The reduced risk of cancer death after surgical and systemic treatments is consistent with the parameters reported within the NSW analysis of all female breast cancers [19]. Direct comparison of (sub) hazard ratios is not possible because of the models derived but the estimated effects of risk reduction from partial and simple mastectomy are more readily compared. Our results align with those of NSW where, after taking account of age, year of diagnosis and stage at diagnosis, partial or localised surgery was associated with greater risk reductions (SHR = 0.10 95%CIs 0.01–0.88 in SA and HR = 0.17 95%CIs 0.15–0.19 in NSW) than simple or complete mastectomy (SHR = 0.23 95%CIs 0.06–0.88 in SA and HR = 0.31 95%CIs 0.28–0.34 in NSW).

The lower utilisation of treatment modes observed among Aboriginal women compared to non-Aboriginal women in our study is also generally consistent with international studies from New Zealand [10, 14]. Simple mastectomy was reported in approximately 45% of Maori women and 34% of non-Maori with breast cancer which aligns closely to the 44% among Aboriginal and 33% non-Aboriginal women in our study. Reports of breast conserving surgery were also consistent among non-Maori (52%) and non-Aboriginal women (49%). Among Aboriginal women however, breast conserving surgery was less common than observed among Maori women (26% versus 44%). Our findings are also consistent with those from NSW, another Australian jurisdiction reporting variations in exposure to surgical treatments [19]. Among non-Aboriginal women with breast cancer in NSW and South Australia, 49% received breast conserving surgery while simple mastectomy was performed in 39% of NSW and 33% of South Australian cases. Fewer Aboriginal women with cancer had breast conserving surgery in South Australia compared to NSW (26% versus 37%) but the relative frequency of mastectomy was similar at 44% and 48% respectively.

Mammography through BSSA is freely available to women aged 40 to 74 years in Australia with active invitation of those aged 50 to 69 during the study period. Equivalent numbers of Aboriginal and non-Aboriginal women in the latter age range were listed by BSSA, yet fewer Aboriginal women participated in screening. Women aged 40 to 49 years are free to initiate contact with BSSA. Relatively few Aboriginal women with breast cancer in this age range had done so, compared to over half the non-Aboriginal women. The results suggest two areas of response are warranted. The first is to continue to innovate in delivering screening to all women, particularly to Aboriginal and Torres Strait Islander women, with the aim of increasing uptake of BSSA invitations and ensuring the screening experience is as favourable as possible for Aboriginal and Torres Strait Islander women [16, 36]. This will include careful consideration of cultural appropriateness in screening activities [17, 37, 38]. The second may be to consider broadening active invitation to include Aboriginal and Torres Strait Islander women aged 40 to 49, since this group represented 25% of breast cancers detected in the cohort. This would require discussion of the appropriateness and effectiveness of mammography for women of these ages and ethnicity [17]. For example, emerging evidence of lower breast density among younger, Aboriginal women [1] and the potential for more aggressive breast cancers in younger women generally [39], may suggest a need to reconsider the sensitivity and specificity of mammography among younger women. This will also challenge current understandings of screening cost and effectiveness and presents an opportunity to revise these models toward equity weighted approaches [40]. Nonetheless, the results suggest an unmet health need whereby more than one-quarter of Aboriginal cases were not actively offered a service specifically intended to reduce risk of cancer death.

Clear disparities in the uptake of cancer treatments were also evident, despite Australia’s health care being universally available. In Australia, equity is a key performance issue in health care delivery [41] and is assessed in terms of timely access to services by special needs groups [42]. Our results provide evidence of inequitable access with Aboriginal cases 65% less likely to be hospitalised with a primary diagnosis of cancer within the observed period, compared to matched, non-Aboriginal cases. Where hospitalisation occurred, further inequities of utilisation and quality [43, 44] emerged, with fewer Aboriginal women with breast cancer treated with systemic therapies, surgery or radiotherapy. There could be biological reasons, dictated by the breast cancer subtypes among Aboriginal women that influence differences in relevant systemic treatments using hormonal tablets, chemotherapy and anti HER2 targeted therapies for example. While valuable, that line of analysis is outside the scope of our current study. Our results make clear that the pervasiveness and extent of observed differences in cancer interventions indicate the need for effective, systematic responses to address these gaps in service access, utilisation and quality. The responses can be informed by systematically attending to patient experiences [45] as a means of promoting improved communication, cultural competency and collaboration among patients and clinicians. Additionally, continued improvements to electronic patient care records can flag system inputs which are effective influences on outputs and patient outcomes. For example, where treatment pathways are initiated, documenting issues relating to patient refusal and clinical contra-indications will delineate obstacles to treatment uptake, where they occurred in the care pathway, and who they involved.

As with previous studies in this subject area [18, 46], we acknowledge our study was limited in its ability to perfectly match on confounding variables included in the initial design. For example, this resulted in the cohort of Aboriginal women being slightly younger on average than their non-Aboriginal contemporaries, but not to a statistically significant extent. Similarly, during the study’s time frame, considerable changes in cancer care practices took place. While differences in year of diagnosis were limited to five-years, we acknowledge it is possible that some residual confounding may have occurred despite our adjusting for the matched variables. Our analysis was strengthened by including a broad range of data collections, using an efficient fixed effects design and accounting for competing risk from non-cancer death. However, low case numbers limited our ability to include ecological associations of geographic remoteness and the influence of comorbid conditions diagnosed among individual cases. We also acknowledge the potential for lead-time effects, and possibly some over diagnosis, leading to an to overestimation of survival improvement associated with breast screening as found in the Australian BreastScreen service evaluations [47]. Nonetheless, our estimates of the risk of cancer death are consistent with outcome studies in the international and domestic literature using administrative hospital records. We also broadened the method used in other outcome studies by including pharmaceutical benefits and Medicare records. Additionally, the CanDAD project successfully piloted the inclusion of breast screening records into a quantitative description of the aetiology of Aboriginal women’s experience with breast cancer. In doing so we began to explore the relationship between cancer outcomes, screening behaviour and treatment exposure, as a recognised major omission in our knowledge base in cancer control [8].

The study therefore enhances our understanding of the risk of breast cancer death among Aboriginal South Australians. Given the CanDAD project’s governance includes Aboriginal community representatives, South Australian Cancer Services and colocation with the South Australian Academic Health Science and Translation Centre, the knowledge developed is directly available for translation into benefits for Aboriginal and Torres Strait Islander people through system-wide cancer control [48] and chronic disease [49] initiatives.

The results alert service planners to the lower levels of screening and treatment of South Australian Aboriginal women with breast cancers and its likely adverse effect on cancer outcomes. This is important evidence for introducing corrective initiatives aimed at earlier detection of cancers and the timely access and uptake of effective cancer treatments, while monitoring health system change to maximise equitable outcomes.

While the findings demonstrate the value of using existing administrative records to assess exposure to, and the outcomes from, components of cancer care from detection to treatment, the data do not inform on re-screening or post-screening assessment [16], time to treatment, whether treatment courses were completed, or the curative intent of interventions. Consequently, our study raises two particular research and development opportunities. The first is to better understand biological influences on age at diagnosis and breast cancer subtypes among Aboriginal women and their subsequent influences on effective treatment modalities. The second is to incorporate screening records for other conditions (bowel and cervix) [8], broaden the screening data fields and further relevant clinical information with the aim of informing continuous quality improvement activities.

## Conclusions

Our results demonstrate the risk of cancer death after breast cancer diagnosis is significantly influenced by controllable factors including: participation in mammographic screening; early stage diagnosis; and treatment with systemic therapies and surgery. Moreover, they demonstrate Aboriginality does not need to be synonymous with poor outcomes following breast cancer diagnosis. Rather, improving exposure to effective interventions among Aboriginal and Torres Strait Islander breast cancer cases is likely to result in improved, more equitable outcomes. Encouraging targeted, earlier access to cancer care services and maximising the utilisation and quality of effective, culturally appropriate cancer interventions in early detection and treatment should be actively promoted to reduce the risk of death from breast cancer and improve survival after breast cancer diagnosis in the Aboriginal and Torres Strait Islander population.

## Abbreviations

95%CIs: 95% confidence intervals
AIC: Akaike Information Criterion
BSSA: BreastScreen SA
CanDAD: Cancer Data and Aboriginal Disparities
IRSAD: Index of Relative Socio-economic Advantage and Disadvantage
NSW: New South Wales
OR: odds ratio
SA: South Australia
SACR: South Australian Cancer Registry
SHR: sub-hazard ratios

## References

[1] Tapia KA (2017). Breast Cancer in Australian indigenous women: incidence, mortality, and risk factors. Asian Pac J Cancer Prev.

[2] Australian Institute of Health and Welfare, Burden of cancer in Australia (2017). Australian Burden of Disease Study 2011.

[3] Banham D (2017). Disparities in cancer stage at diagnosis and survival of aboriginal and non-aboriginal south Australians. Cancer Epidemiol.

[4] Shaw IM, Elston TJ (2003). Retrospective, 5-year surgical audit comparing breast cancer in indigenous and non-indigenous women in far North Queensland. ANZ J Surg.

[5] Seneviratne S, et al. Impact of mammographic screening on ethnic and socioeconomic inequities in breast cancer stage at diagnosis and survival in New Zealand: A cohort study. BMC Public Health. 2015;15(1):46. 10.1186/s12889-015-1383-4 PMC4314740 25637343

[6] Roder D (2017). Breast cancer screening—opportunistic use of registry and linked screening data for local evaluation. J Eval Clin Pract.

[7] Roder D (2008). Population screening and intensity of screening are associated with reduced breast cancer mortality: evidence of efficacy of mammography screening in Australia. Breast Cancer Res Treat.

[8] Australian Institute of Health and Welfare (2018). Analysis of cancer outcomes and screening behaviour for national cancer screening programs in Australia.

[9] Chen L, Li CI (2015). Racial Disparities in Breast Cancer Diagnosis and Treatment by Hormone Receptor and HER2 Status. Cancer Epidemiol Biomarkers Prev.

[10] Tin Tin S (2018). Ethnic disparities in breast cancer survival in New Zealand: which factors contribute?. BMC Cancer.

[11] Seneviratne S (2014). Ethnic differences in timely adjuvant chemotherapy and radiation therapy for breast cancer in New Zealand: a cohort study. BMC Cancer.

[12] Seneviratne S (2015). Ethnic differences in breast cancer survival in New Zealand: contributions of differences in screening, treatment, tumor biology, demographics and comorbidities. Cancer Causes Control.

[13] Seneviratne S (2015). Treatment delay for Māori women with breast cancer in New Zealand. Ethn Health.

[14] Seneviratne S (2017). Ethnic, socio-demographic and socio-economic differences in surgical treatment of breast cancer in New Zealand. ANZ J Surg.

[15] Australian Institute of Health and Welfare (2017). BreastScreen Australia monitoring report 2014–2015.

[16] Roder D (2012). Breast screening and breast cancer survival in aboriginal and Torres Strait islander women of Australia. Asian Pac J Cancer Prev.

[17] Dasgupta P (2017). Variations in outcomes for indigenous women with breast cancer in Australia: a systematic review. Eur. J Cancer Care.

[18] Moore SP (2016). Breast cancer diagnosis, patterns of care and burden of disease in Queensland, Australia (1998–2004): does being indigenous make a difference?. Int J Public Health.

[19] Supramaniam R (2014). Increasing rates of surgical treatment and preventing comorbidities may increase breast cancer survival for aboriginal women. BMC Cancer.

[20] Yerrell Paul Henry, Roder David, Cargo Margaret, Reilly Rachel, Banham David, Micklem Jasmine May, Morey Kim, Stewart Harold Bundamurra, Stajic Janet, Norris Michael, Brown Alex (2016). Cancer Data and Aboriginal Disparities (CanDAD)—developing an Advanced Cancer Data System for Aboriginal people in South Australia: a mixed methods research protocol. BMJ Open.

[21] Banham D, Brown A, Roder D (2018). Comorbidities contribute to the risk of cancer death among aboriginal and non-aboriginal south Australians: analysis of a matched cohort study. Cancer Epidemiol.

[22] Buckley ES (2016). The utility of linked cancer registry and health administration data for describing system-wide outcomes and research: a BreastScreen example. J Eval Clin Pract.

[23] Wardliparingga Aboriginal Research Unit (2014). South Australian Aboriginal Health Research Accord: Companion Document.

[24] Fritz A (2000). International Classification of Diseases for Oncology.

[25] Young JJ (2001). SEER Summary Staging Manual - 2000: Codes and Coding Instructions.

[26] Australian Bureau of Statistics (2013). Socio-Economic Indexes for Areas (SEIFA) - Technical Paper, 2011.

[27] Elsworthy A (2013). Australian classification of health interventions: ACHI: Alphabetic index of interventions.

[28] Sax Institute (2016). Secure Unified Research Environment (SURE).

[29] StataCorp, Stata Statistical Software: Release 14.2. 2015, StataCorp LP: College Station, TX.

[30] O’Keefe CM (2008). Privacy and the Use of Health Data - Reducing Disclosure Risk. electronic Journal of Health Informatics.

[31] National Health Services Scotland (2010). ISD Statistical Disclosure Control Protocol.

[32] Akinyemiju TF (2018). Demographic, presentation, and treatment factors and racial disparities in ovarian cancer hospitalization outcomes. Cancer Causes Control.

[33] Fine JP, Gray RJ (1999). A proportional hazards model for the subdistribution of a competing risk. J Am Stat Assoc.

[34] Akaike H (1974). A new look at the statistical model identification. IEEE Trans Autom Control.

[35] Schoenfeld D (1982). Partial residuals for the proportional hazards regression model. Biometrika.

[36] Cancer Australia. Study of breast cancer screening characteristics and breast cancer survival in Aboriginal and Torres Strait Islander women of Australia. Surry Hills: Cancer Australia; 2012.

[37] Pilkington L (2017). Perspectives of aboriginal women on participation in mammographic screening: a step towards improving services. BMC Public Health.

[38] Reilly Rachel, Micklem Jasmine, Yerrell Paul, Banham David, Morey Kim, Stajic Janet, Eckert Marion, Lawrence Monica, Stewart Harold B., Brown Alex (2018). Aboriginal experiences of cancer and care coordination: Lessons from the Cancer Data and Aboriginal Disparities (CanDAD) narratives. Health Expectations.

[39] Paluch-Shimon S (2017). ESO-ESMO 3rd international consensus guidelines for breast cancer in young women (BCY3). Breast.

[40] Baeten SA (2010). Incorporating equity-efficiency interactions in cost-effectiveness analysis-three approaches applied to breast cancer control. Value Health.

[41] Allin S, Hernandez-Quevedo C, Massiera C, Smith PC (2009). Measuring equity of access to health care, in Performance measurement for health system improvement: Experiences, challenges and prospects.

[42] SCRGSP (steering Committee for the Review of government service provision), Report on Government Services 2018. 2018, Productivity Commission: Canberra.

[43] Whitehead M (1990). The concepts and principles of equity and health.

[44] Liu JB, Ko CY (2017). Disparities in rectal Cancer: moving from descriptions to solutions. Ann Surg Oncol.

[45] Wotherspoon C, Williams CM. Exploring the experiences of Aboriginal and Torres Strait Islander patients admitted to a metropolitan health service. Aust Health Rev. 2018;43(2);217–23. 10.1071/AH17096 29495978

[46] Valery P (2006). Cancer diagnosis, treatment, and survival in indigenous and non-indigenous Australians: a matched cohort study. Lancet.

[47] Roder D (2014). Breast cancer screening: update in Australian context. Cancer Forum.

[48] SA Health. South Australian Aboriginal Cancer Control Plan 2016–2021. Adelaide: Government of South Australia; 2016.

[49] Keech W (2017). South Australian Chronic Disease Consortium Road Map 2017-2021.

[50] Farshid G, Roder D (2015). Integrating BreastScreen SA screening records into the Advanced Cancer Data System (ACaDS) pilot.

